# Supplementary material for: Temperament and character profiles of medical students associated with tolerance of ambiguity and perfectionism
Source: PeerJ. 2019 Jun 13;7:e7109. doi: 10.7717/peerj.7109 (PMC6571128; doi:10.7717/peerj.7109)
Supplement: Supplemental Information 1 — Supplementary details on definition and methods on the Temperament and character profiles. [file peerj-07-7109-s001.docx]

## Supplementary Materials

#

# Appendix A: High and low interpretations and representations for each of the seven temperament and character traits.

| **Temperament traits** | **Represents** | **LOW SCORES** |  | **HIGH SCORES** |
| --- | --- | --- | --- | --- |
| Novelty Seeking | Exploratory activity in response to novelty | Orderly, reflective, reserved | **<--->** | Exploratory, curious, |
|  |  |  |  | seeks challenge |
| Harm Avoidance | Worry in anticipation of problems | Confident, accepting of uncertainty & risk | **<--->** | Anxious, uncomfortable |
|  |  |  |  | with accepting risk |
| Reward Dependence | Dependence on approval of others | Not influenced by others, objective, insensitive | **<--->** | Needs to please, warm, attached, sociable |
| Persistence | Industriousness of behaviour despite obstacles | Quitting, underachiever, erratic, unambitious | **<--->** | Ambitious, hard worker, diligent, |
|  |  |  |  | perfectionist |
| **Character** | **Represents** | **LOW SCORES** | **<--->** | **HIGH SCORES** |
| **traits** |  |  |  |  |
| Self-Directedness | Responsibility, goal orientated & self-confidence | Blaming, ineffective, unreliable, irresponsible | **<--->** | Conscientious, |
|  |  |  |  | self-accepted, |
|  |  |  |  | reliable, |
| Cooperativeness | Tolerance, cooperativeness & empathy | Intolerant, unhelpful opportunistic, | **<--->** | Tolerant, agreeable, constructive, |
|  |  | critical |  | empathic |
| Self-Transcendence | View of self in relation to the universe as a whole | Impatient, proud, materialistic, practical | **<--->** | Patient, humble, |
|  |  |  |  | spiritual, creative, compassionate |
| *Note.* Adapted from Cloninger et al, 1994 [29] | | | | |

# Appendix B: Details on the classification methods of the two personality profiles based on the seven TCI subscales.

To identify profiles of individuals with distinctive combinations of the seven traits, a latent profile analysis (LPA) was conducted on the trait scores using MPlus 6.12. The LPA identified sub-groups (profiles) within the sample based on similarity of responses to the 7 trait scores. The analysis was conducted by comparing multiple profiles with a one-profile solution, adding more profiles until the additional one no longer yielded a significant improvement in model fit statistics. Determination of the number of profiles was based on a number of fit criteria. Good model fit indices were low Akaike information criterion (AIC), Bayesian information criterion (BIC) and sample size adjusted BIC. A lower value of these criteria indicates a better balance of model parsimony and model fit. Entropy was used to measure the classification uncertainty from 0 to 1. Higher values indicated clearer classification. A significant Lo-Mendell-Rubin likelihood ratio test (LMR-LRT) indicated that the current model had a significant better fit than the previous model (with one less profile). Results of the LPA fit indices supported a two-profile solution (see Table B1). The LMR-LRT indicated that the two-profile model fit the data significantly better than the one profile model (p < 0.001) and not worse than a three-profile model (p = 0.344). Entropy was also higher in the two-profile solution than it was in the three-profile solution, which indicated that the two-profile solution had a clearer profile distinction. The two-profile solution was used to define personality profile in our study based on parsimony and greater entropy; the three-profile solution had lower entropy, only slightly lower BIC, and the LMR test was not significantly better.

## Table B1: Fit indices from the latent profile analysis to determine the classification of personality profiles of medical students based on the seven TCI subscales.

|  | **Profiles specified** | | |
| --- | --- | --- | --- |
| **Fit statistics** | **1** | **2*** | **3** |
| Log likelihood | -3978.77 | -3701.06 | -3635.50 |
| Akaike information criterion | 7985.54 | 7985.54 | 7331.00 |
| Bayesian information criterion | 8051.26 | 7549.39 | 7471.84 |
| Sample size adjusted Bayesian information criterion | 8006.81 | 7479.53 | 7376.57 |
| Entropy | - | 0.71 | 0.66 |
| LMR-LRT, Lo-Mendell-Rubin likelihood ratio test | - | 545.25 | 128.71 |
| p-value | - | <.001 | .344 |
| *Model with best fit statistics | | | |
